# Hyperactive ERK and persistent mTOR signaling characterize vemurafenib resistance in papillary thyroid cancer cells

**DOI:** 10.18632/oncotarget.6779

**Published:** 2015-12-28

**Authors:** Elyse K. Hanly, Neha Y. Tuli, Robert B. Bednarczyk, Robert Suriano, Jan Geliebter, Augustine L. Moscatello, Zbigniew Darzynkiewicz, Raj K. Tiwari

**Affiliations:** ^1^ Department of Microbiology and Immunology, New York Medical College, Valhalla, NY 10595, USA; ^2^ Division of Natural Sciences, College of Mount Saint Vincent, Bronx, NY 10471, USA; ^3^ Department of Otolaryngology/Head and Neck Surgery, New York Medical College, Valhalla, NY 10595, USA; ^4^ Department of Pathology, New York Medical College, Valhalla, New York 10595, USA

**Keywords:** thyroid cancer, vemurafenib, BRAFV600E, drug resistance, cell signaling

## Abstract

Clinical studies evaluating targeted BRAFV600E inhibitors in advanced thyroid cancer patients are currently underway. Vemurafenib (BRAFV600E inhibitor) monotherapy has shown promising results thus far, although development of resistance is a clinical challenge. The objective of this study was to characterize development of resistance to BRAFV600E inhibition and to identify targets for effective combination therapy. We created a line of BCPAP papillary thyroid cancer cells resistant to vemurafenib by treating with increasing concentrations of the drug. The resistant BCPAP line was characterized and compared to its sensitive counterpart with respect to signaling molecules thought to be directly related to resistance. Expression and phosphorylation of several critical proteins were analyzed by Western blotting and dimerization was evaluated by immunoprecipitation. Resistance to vemurafenib in BCPAP appeared to be mediated by constitutive overexpression of phospho-ERK and by resistance to inhibition of both phospho-mTOR and phospho-S6 ribosomal protein after vemurafenib treatment. Expression of potential alternative signaling molecule, CRAF, was not increased in the resistant line, although formation of CRAF dimers appeared increased. Expression of membrane receptors HER2 and HER3 was greatly amplified in the resistant cancer cells. Papillary thyroid cancer cells were capable of overcoming targeted BRAFV600E inhibition by rewiring of cell signal pathways in response to prolonged vemurafenib therapy. Our study suggests that *in vitro* culture of cancer cells may be useful in assessing molecular resistance pathways. Potential therapies in advanced thyroid cancer patients may combine vemurafenib with inhibitors of CRAF, HER2/HER3, ERK, and/or mTOR to delay or abort development of resistance.

## INTRODUCTION

Thyroid cancer incidence in the United States is increasing and patients with metastatic and radioiodine-resistant disease are in need of new treatments. Targeted therapy is a promising option for papillary thyroid cancer cases in which surgery and radioiodine are ineffective. In particular, targeting *BRAFV600E*, a mutation found in over one-half of papillary thyroid cancers and associated with worse prognosis, may provide clinical utility [1–3]. *BRAF* is a human gene coding a proto-oncogene protein called B-Raf which is a serine/threonine kinase. Results of an ongoing phase II clinical study including patients with metastatic or unresectable *BRAFV600E*-positive papillary thyroid cancer demonstrate that vemurafenib (BRAFV600E kinase inhibitor) has an anti-tumor effect when used as a monotherapy (Brose *et al*., 2013 ECCO/ESMO/ESTRO Annual Meeting). These results are consistent with an earlier phase I clinical study, a case study involving a patient with advanced papillary thyroid cancer, and off-label use of vemurafenib (VMR) in 17 patients [4–6].

Early clinical successes of VMR can be attributed to the mechanism of action it was designed for- inhibiting overactive BRAFV600E kinase within the pro-survival MAPK/ERK cell signaling pathway. ERK signaling serves as a link to convert extracellular stimuli into transcriptional programs affecting various processes such as cell growth, differentiation, proliferation, and apoptosis. Dysregulation of the ERK pathway occurs in one-third of all cancers and is mediated by genetic lesions resulting in events such as overexpression or constitutive activation of kinases and/or continuous production of activating ligands [7]. Within papillary thyroid cancer, mutations including *BRAFV600E*, *RET/PTC* and/or *RAS* are found in over 70% of cases. Each mutation contributes to ERK pathway activation and tends to be mutually exclusive within thyroid tumors [8, 9]. The most common mutation, *BRAFV600E,* results in a constitutively active kinase driving ERK signaling and thyroid cancer progression. Presence of the *BRAFV600E* mutation has been correlated with recurrence, resistance to radioiodine, and increased mortality [3, 10–13]. Inhibition of BRAFV600E kinase with VMR within thyroid cancer cells has been shown to inhibit cell proliferation and induce apoptosis [14–16].

A problem to overcome in the treatment of patients with small molecule inhibitors such as VMR is the development of drug resistance due to the adaptability of most cancers [17, 18]. Cancers treated with a single specific inhibitor of a ERK pathway protein will inevitably lose responsiveness to the drug and continue to express activated ERK [19]. The development of adaptive resistance can began shortly after treatment in cancer cells due to sudden inhibition of signaling leading to changes in feedback mechanisms. A signal rewiring is initiated and will persist if successful. It is also generally accepted that certain cells within a solid tumor are better able to accommodate the presence of a targeted inhibitor, perhaps due to a mutation or availability of alternative signaling proteins or pathways. Such cells will be clonally selected for in an evolutionary way after treatment with the inhibitor leading to drug-resistance of the tumor [17].

Based on data in literature and our prior findings, we hypothesize that determining targets of VMR resistance in thyroid cancer may be useful in developing rational combination therapy that limits resistance or exploits susceptibilities of resistant tumors. Successful clinical trials of VMR in melanoma patients demonstrated improved survival, although resistance often developed months after treatment [20, 21]. A number of studies found that resistance in these cases occurred mainly through reactivation of ERK signaling [22–31]. ERK reactivation can be propagated by increased expression or activity of upstream signaling molecules such as receptor tyrosine kinases (RTKs) in response to VMR treatment [32–34]. In addition, studies have attributed resistance to increased expression of kinases COT and CRAF, which are capable of contributing to ERK signaling independent of BRAF protein [22, 23]. It is expected that inhibitors of the kinases shown to be activated by persistent treatment with VMR, when used in combination therapy, may delay or abolish resistance to VMR.

There are few studies which examine possible resistance mechanisms to BRAFV600E inhibition in thyroid cancer. One group found that VMR treatment led to increased transcription and expression of HER3 receptor in thyroid cancer cells. These cells could be sensitized to BRAF inhibition with the addition of HER kinase inhibitor lapatinib [35]. In our study, we created resistance to VMR *in vitro* using BCPAP papillary thyroid cancer cells. We then analyzed differences in expression of ERK pathway signaling molecules in normal thyroid cells, papillary thyroid cancer cells, VMR-resistant papillary thyroid cancer cells, and inherently-resistant anaplastic thyroid cancer cells. Signaling molecules in the study included CRAF, HER2, HER3, ERK, and mTOR. We hypothesized that resistance develops through reactivation of ERK, mediated by increased upstream signaling and activation of molecules providing signaling alternatives to BRAFV600E. The goal of the present study was to identify target molecules that may contribute to VMR resistance in thyroid cancer cells. Further studies in which VMR is included in combination therapy may greatly benefit *BRAFV600E*-positive advanced thyroid cancer patients.

## RESULTS

### *In vitro* resistance to vemurafenib develops in BCPAP cells

**Figure 1:**
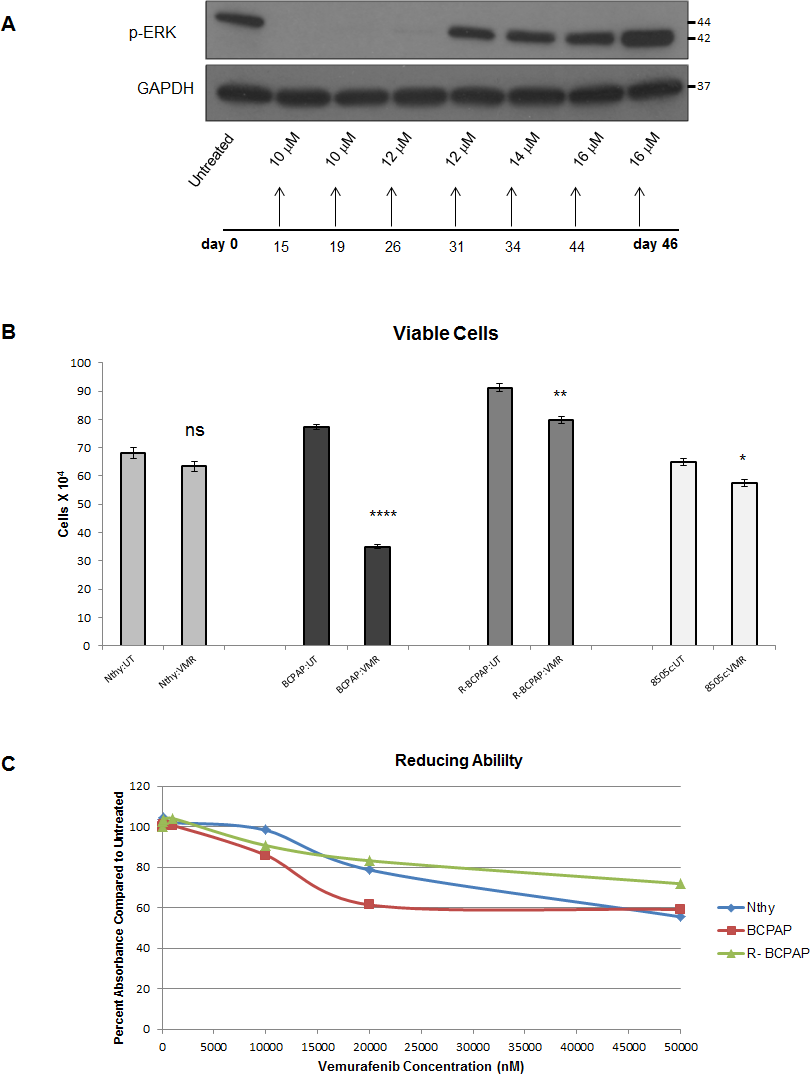
*In vitro* resistance to vemurafenib develops in BCPAP cells (**A**) Cell lysates from BCPAP cells were collected after 24 hour treatment periods with gradually increasing concentrations of vemurafenib (VMR), subjected to SDS-PAGE and analyzed by Western blot for expression of phospho-ERK1/2 at the end of the 45 day experiment. (**B**) BCPAP and resistant BCPAP cells (from A) were treated with 10 μM VMR for 36 hours and viable cells were counted by trypan blue exclusion assays. ****P <.0001. (**C**) Nthy-ori 3-1 normal thyroid cells, BCPAP, and resistant BCPAP cells were treated with varying concentrations of VMR for 96 hours and then incubated with Alamar blue for 4 hours. Percent absorbance is representative of the difference between measured absorbance at 570 nm and the control absorbance at 600 nm. Data in Figure 1B was published previously in Oncotarget. 2015; 6:39702–13.

We investigated resistance to BRAFV600E inhibition *in vitro* by treating *BRAFV600E*-positive papillary thyroid cells (BCPAP) with increasing concentrations of VMR (BRAFV600E inhibitor). Cells were initially susceptible to this drug and showed a lack of detectable phospho-ERK after 24 hour treatment, thereby demonstrating inhibition of signaling. Cells began to express detectable levels of phospho-ERK after the subsequent 12 μM treatment period and continued to show expression throughout the rest of the experiment, after treatment up to 16 μM VMR (Figure 1A). Reappearance of phospho-ERK indicates that cells adapted to activate ERK despite the VMR-mediated block of BRAFV600E. These conditioned cells (resistant BCPAP) were maintained in culture and subjected to proliferation assays. Resistant BCPAP cells demonstrated diminished growth-inhibitory effects in response to VMR compared to BCPAP in our prior study (Figure 1B) [36]. In addition, an Alamar blue viability assay supported these results, since resistant cells maintained higher ability to reduce resazurin (an indicator of cell viability) compared to normal BCPAP cells over a range of vemurafenib concentrations (Figure 1C). At greater concentrations of VMR, the resistant BCPAP cells surpassed *BRAF*-wild-type Nthy-ori 3-1 cells in terms of viability (Figure 1C).

### CRAF expression is not directly modulated by vemurafenib and resistance may be characterized by increased CRAF dimer formation

**Figure 2:**
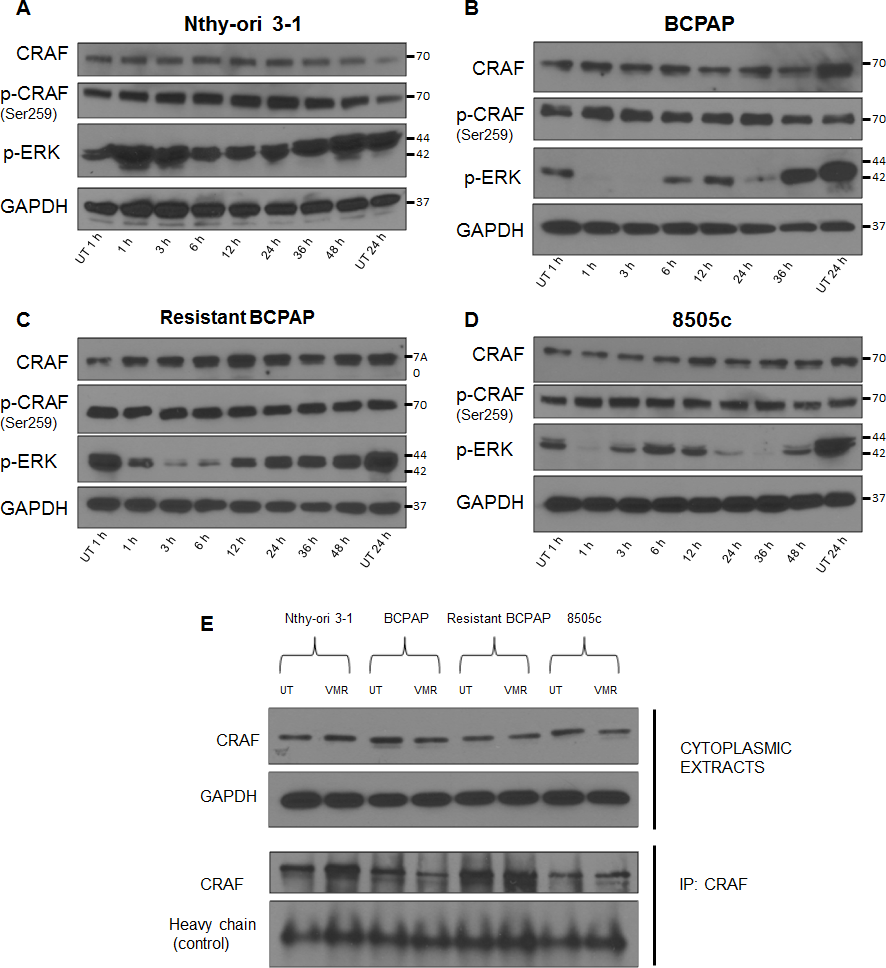
Resistant BCPAP cells do not exhibit oscillatory inhibition of phospho-ERK and have increased CRAF dimers (**A–D**) Nthy-ori 3–1, BCPAP, 8505c, and resistant BCPAP cells were subjected to 10 μM VMR for various time points and cytoplasmic extracts were collected and analyzed by Western blot for expression of phospho-ERK1/2, CRAF, phospho-CRAF, and GAPDH (loading control). (**E**) Cytoplasmic extracts from thyroid cell lines were collected after 24 hours untreated and treated with 10 μM VMR and analyzed by Western blot for comparison of CRAF expression. After being subjected to the same 24 hour treatment, thyroid cell lines were collected and lysed using 1% NP-40 buffer. Lysates were subjected to immunoprecipitation using anti-CRAF antibody, SDS-PAGE, and Western blot analysis for CRAF.

Within the ERK signaling pathway, CRAF is a wild-type RAF isotype, and can transmit signals from GTP-bound RAS to MEK, which activates ERK. In this classical RAS-dependent kinase cascade, it is thought that dimer formation of CRAF is necessary to propagate signaling, which is not the case for mutated BRAFV600E signaling [37]. Since increased CRAF expression was shown to contribute to VMR resistance in melanoma, we analyzed CRAF expression and dimer formation in our thyroid cancer cells in response to this drug. Thyroid cells were treated with 10 μM VMR and collected at various time points including 1, 3, 6, 12, 24, 36, and 48 hours. Cell lines included *BRAF*-wild-type normal thyroid cells (Nthy-ori 3-1), *BRAFV600E*-positive papillary thyroid cancer cells (BCPAP), VMR-resistant BCPAP, and *BRAFV600E*-positive anaplastic thyroid cancer cells (8505c). Throughout treatment, CRAF expression and phosphorylation remained constant in the four thyroid cell lines, indicating that expression is not directly modulated by VMR (Figure 2A–2D).

To compare total CRAF expression among the thyroid cell lines and to determine if the expression of CRAF changes in response to VMR after resistance, we included all four cell lines on a Western blot after 24 hours of treatment. We found no difference in overall CRAF expression among cell lines (Figure 2E). However, when an immunoprecipitation experiment was conducted using an anti-CRAF antibody to extract protein complexes from cell lysates, a greater amount of CRAF was isolated from VMR-resistant cells compared to normal BCPAP (Figure 2E). This data indicates that although total CRAF levels do not change, the formation of CRAF dimers may be increased in VMR-resistant BCPAP cells. Since activation of upstream RAS promotes RAF dimerization, we predicted that the increase in CRAF dimers occurred as a result of enhanced upstream signaling due to loss of negative feedback by ERK. In normal Nthy-ori 3-1 thyroid cells, an increase in CRAF dimers in response to 24 hour VMR treatment was observed, which may reflect the RAF inhibitor paradox, in which wild-type RAF dimer interactions are enhanced (Figure 2E).

### BCPAP cells are sensitive to oscillatory inhibition of phospho-ERK by vemurafenib and resistant BCPAP cells are not

Interestingly, treating thyroid cells with VMR over various time points revealed an oscillatory pattern of ERK inhibition in BCPAP and 8505c. Inhibition occurred early at 1–3 hours and then phospho-ERK expression rebounded around 6 hours before inhibition ensued at 24 hours (Figure 2B, 2D). This phenomenon has been reported in the literature and was attributed to expression kinetics of DUSP5, an ERK phosphatase regulated by ERK activation [35, 38]. VMR-resistant BCPAP cells and *BRAF*-wild-type Nthy-ori 3-1 cells did not express this oscillatory pattern (Figure 2A, 2C). The resistant BCPAP line demonstrated only early inhibition of ERK and then remained uninhibited throughout the 48 hour treatment (Figure 2C). In contrast, normal BCPAP demonstrated oscillatory inhibition and cells were unable to survive treatment up to 48 hours (Figure 2B). The difference in expression pattern of phospho-ERK in response to VMR supports a role for ERK re-activation in adaptive resistance. However, sensitivity to oscillatory inhibition of phospho-ERK is not necessarily correlated with growth inhibition. When analyzing the anaplastic line (8505c), we found that these cells exhibit oscillatory ERK inhibition without detrimental effect on cell growth, suggesting pre-existing alternative signaling circuits in these cells (Figure 2D).

### Expression of receptor tyrosine kinases, HER2 and HER3, is increased in vemurafenib-resistant BCPAP cells

**Figure 3:**
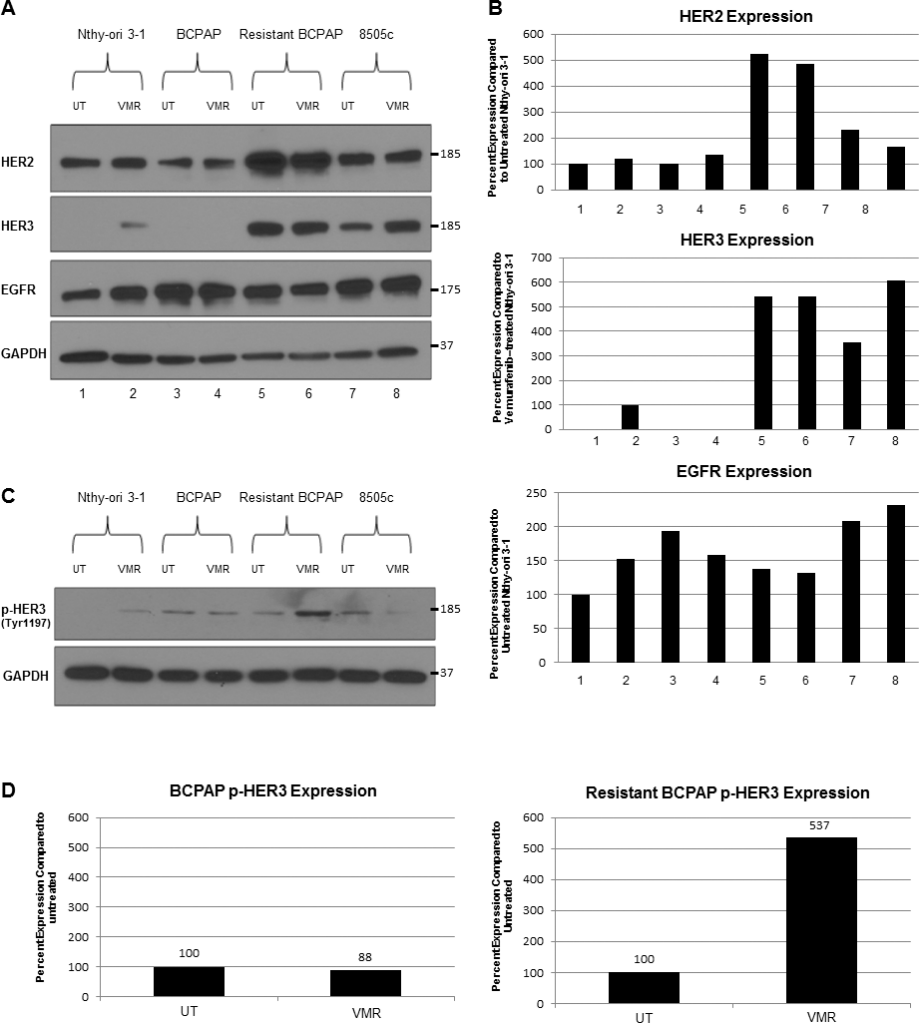
Resistant BCPAP cells are characterized by increased expression of HER2 and HER3 and increased HER3 phosphorylation in response to vemurafenib (**A**) Whole cell lysates from thyroid cell lines were collected after 24 hours +/− 10 μM VMR, subjected to SDS-PAGE, and analyzed by Western blot for expression of HER2, HER3, EGFR, and GAPDH (loading control). (**B**) Densitometry of Western blots in A was performed using ImageJ and expressed as percent expression compared to Nthy-ori 3–1 after normalizing each sample to GAPDH. (**C**) Cell lysates were collected as in A and analyzed for expression of phospho-HER3 (Tyr1197) and GAPDH as a loading control. (**D**) Densitometry of Western blots in C was performed using ImageJ and expressed as percent expression compared to untreated for BCPAP and resistant BCPAP samples.

To further investigate the role of feedback inhibition release caused by VMR, we analyzed expression of upstream membrane receptors by Western blot (Figure 3). We found that the resistant BCPAP cells expressed increased amounts of total HER2 compared to susceptible BCPAP cells (Figure 3A, 3B). BCPAP cells did not express detectable levels of HER3 protein in this experiment and the resistant BCPAP cells expressed relatively greater amounts. In addition, the resistant BCPAP cells responded to 24 hour VMR treatment by increasing phosphorylation of HER3 compared to untreated (Figure 3C). HER3 lacks tyrosine kinase activity itself, but becomes phosphorylated at tyrosine residues after forming heterodimers with other ErbB proteins such as HER2. The HER2/HER3 dimer is a potent activator of growth signaling pathways including MAPK. An intermediate expression level was seen in 8505c, as these cells expressed more HER2 and HER3 compared to BCPAP but less compared to resistant BCPAP. Expression of EGFR, which belongs to the same protein receptor family as HER2 and HER3, decreased slightly in the resistant BCPAP cells compared to normal BCPAP.

### Hyperactivation of ERK and resistance to phospho-mTOR inhibition characterize *in vitro* vemurafenib resistance in BCPAP cells

**Figure 4:**
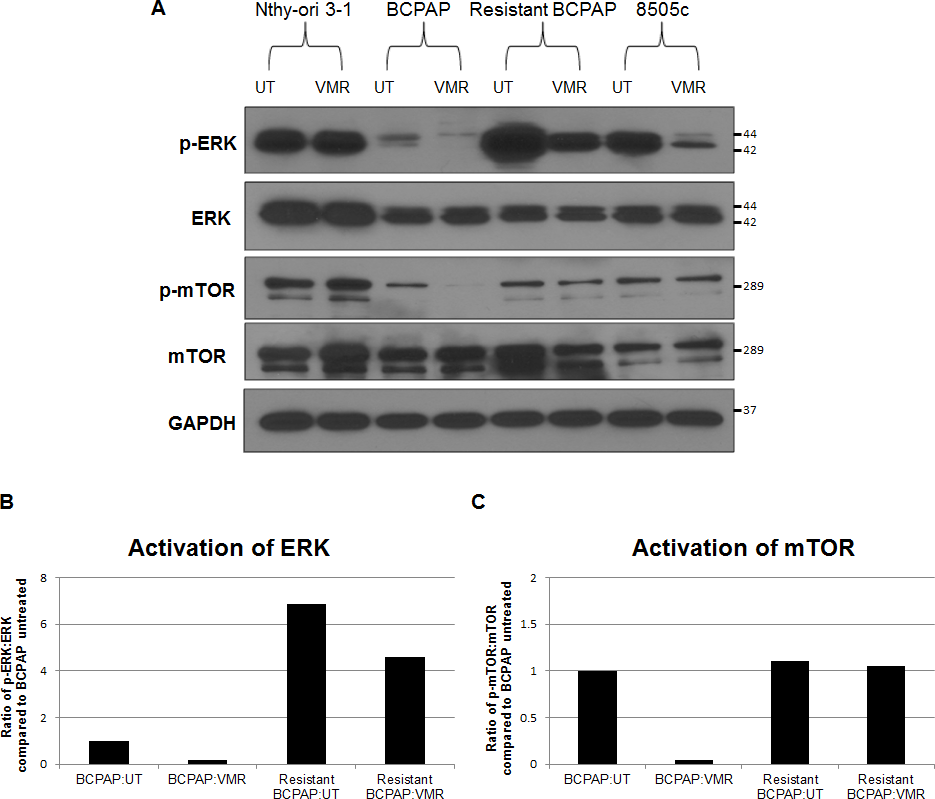
Vemurafenib resistance is mediated by hyperactivation of ERK and loss of vemurafenib-mediated phospho-mTOR inhibition (**A**) Cytoplasmic extracts from thyroid cell lines were collected after 24 hours +/− 10 μM VMR, subjected to SDS-PAGE, and analyzed by Western blot for expression of phospho-MEK, MEK, phospho-ERK1/2, ERK1/2, phospho-mTOR, and mTOR. (**B**) Activation of ERK1/2 was analyzed by calculating the ratio of phospho-ERK1/2 to ERK1/2 after normalizing to GAPDH and using ImageJ for densitometry. (**C**) Activation of mTOR was analyzed the same way as ERK in B.

Since development of resistance appeared to be mediated through mechanisms involved in ERK signaling, we analyzed expression of key effector molecules, ERK and mTOR. We noted inhibition of phosphorylation of these molecules in normal BCPAP cells in response to 24 hour VMR treatment in a previous study [16]. When comparing expression levels of phosphorylated signaling proteins among cell lines, we found that the resistant BCPAP cells expressed a remarkably higher amount of phospho-ERK compared to normal BCPAP (Figure 4). VMR treatment decreased phospho-ERK expression in both normal and resistant BCPAP cells, although the detected amount of phospho-ERK in VMR-treated resistant BCPAP was still greater than was seen in untreated BCPAP cells (Figure 4A). Resistant cells also had higher ratios of phospho-ERK to total ERK, indicating increased constitutive signal activation (Figure 4B). Normal BCPAP cells demonstrated phospho-mTOR inhibition and a decreased ratio of phospho-mTOR to mTOR in response to VMR. However, the resistant BCPAP cells expressed an equal ratio of phospho-mTOR to total mTOR in untreated and VMR-treated samples, indicating resistance to VMR-mediated inhibition (Figure 4A, 4C).

### Resistant BCPAP are relatively resistant to vemurafenib-mediated inhibition of phospho-S6 ribosomal protein compared to susceptible BCPAP

**Figure 5:**
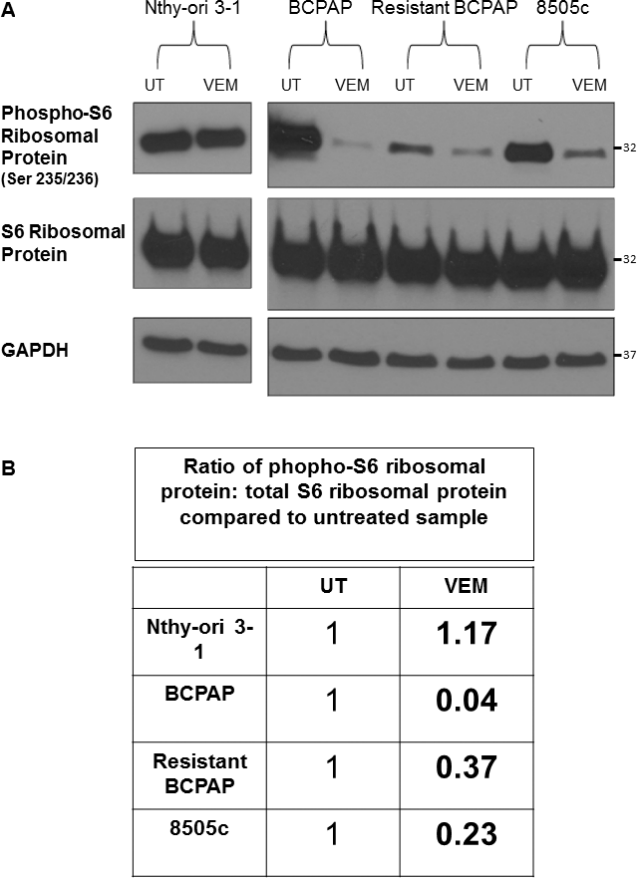
Resistant BCPAP cells are less sensitive to vemurafenib-mediated inhibition of phospho-S6 ribosomal protein compared to BCPAP (**A**) Cytoplasmic extracts from thyroid cell lines were collected after 24 hours +/− 10 μM VMR, subjected to SDS-PAGE, and analyzed by Western blot for expression of phospho-S6 ribosomal protein and S6 ribosomal protein. (**B**) Activation ratios of S6 ribosomal protein were calculated based on densitometry and normalized to the loading control and the untreated sample for each cell line.

Both ERK and mTOR signaling have been shown to contribute to the activation of molecules regulating translation, such as ribosomal S6 kinase (S6K) and its downstream substrate S6 ribosomal protein [39, 40]. We therefore investigated effects of VMR on S6 ribosomal protein expression and its activation in our VMR-sensitive and VMR-resistant thyroid cell lines. Results demonstrated that VMR treatment reduced the phospho-S6 ribosomal protein to total S6 ribosomal protein ratio more drastically in the susceptible BCPAP compared to resistant BCPAP (Figure 5A, 5B). Activation ratios of S6 ribosomal protein decreased to only 0.04 relative to untreated in BCPAP and decreased to 0.37 of the untreated ratio in resistant BCPAP, although BCPAP expressed greater amounts of phospho-S6 ribosomal protein compared to resistant BCPAP in the untreated samples (Figure 5A, 5B). The relative ratio of S6 ribosomal activation after VMR treatment for 8505c anaplastic cells was 0.23 compared to untreated, indicating that the resistant BCPAP more effectively resisted inhibition of phospho-S6 ribosomal protein even compared to 8505c. The *BRAF*-wild-type thyroid cells (Nthy-ori 3-1) had a slightly increased ratio of phospho-S6 ribosomal protein to total S6 ribosomal protein after VMR treatment.

## DISCUSSION

In this study, we characterized cellular resistance to BRAFV600E inhibition in BCPAP papillary thyroid cancer cells in terms of signaling proteins. A main feature of resistant cells was hyperactivation of ERK signaling as demonstrated most clearly by increased expression of phospho-ERK and an increased ERK activation ratio compared to normal BCPAP (Figure 4A, 4B). Our data suggest that mechanisms contributing to enhanced ERK signaling may include increased expression of upstream HER2 and HER3 receptors (Figure 3) and formation of CRAF dimers (Figure 2E). Such activation leads to the signaling that bypasses BRAFV600E within this pathway. In addition, resistant cells appeared to be less vulnerable to inhibition of mTOR activation (Figure 4A, 4C) and inhibition of S6 ribosomal protein activation (Figure 5) by VMR. Investigating the capability of thyroid cancer cells to develop resistance to targeted BRAFV600E *in vitro* may lead to greater understanding of the mechanisms available to thyroid cancer cells within patient tumors and direct future aims to prevent or treat resistance through combination therapies.

Our experiments involved forcing cells in culture to adapt to increasing concentrations of the targeted inhibitor. Although this rather short-term *in vitro* model of inducing VMR resistance may be seen as a limitation, our results exhibit concordance with prior *in vitro* and *in vivo* studies. For example, one of the most common mechanisms of adaptive resistance to targeted inhibitors is rebound expression of receptor tyrosine kinases (RTKs) as a result of loss of feedback inhibition from ERK [33, 41–42]. In our VMR-resistant thyroid cancer cells, we observed both increased expression of the membrane receptors HER2 and HER3 and increased activation of HER3 in response to VMR. These results are consistent with the observation that short-term BRAFV600E inhibitor treatment also induced expression and activation of HER2/HER3 dimers in *BRAF*-mutant thyroid cancer cells [35]. Mechanisms contributing to ERK activation in the presence of pathway inhibitors are likely complex and cell-specific. The identification of potential drug targets that are characteristic of certain types of cancer, such as HER2/HER3 in papillary thyroid cancer, will accelerate the process of developing effective combination therapies. Our study demonstrates that the creation of *in vitro* resistance may be a useful tool to identify resistance molecules for further investigation, especially if this technique is applied more broadly and/or with incorporation of patient-derived models.

Although RAS activity was not measured, we predict that RAS activity increased during development of VMR resistance as a result of the increase in HER2 and HER3 receptors, which dimerize to potently activate RAS. Since activated RAS promotes the dimerization of wild-type RAF proteins, our prediction agrees with the finding of increased CRAF dimers in VMR-resistant BCPAP cells compared to susceptible BCPAP. The formation of CRAF dimers offers a mechanism to directly bypass the inhibited BRAFV600E molecule and continue signaling through the ERK pathway in a BRAFV600E-independent manner.

It is likely that *BRAFV600E*-positive thyroid cancer cells are capable of dynamically altering expression and signaling patterns of cellular proteins in many ways to gain resistance to VMR. The complex interaction of ERK and mTOR signaling in this process is of great interest and requires further study. These two pathways are thought to converge at the regulation of factors involved in translation initiation. ERK signaling activates the p90 ribosomal protein S6 kinases (RSKs) and mTOR signaling activates ribosomal protein S6 kinase (S6K) [41, 42]. Both RSK and S6K phosphorylate S6 ribosomal protein, which then interacts with tRNA, initiation factors, and mRNA [43]. S6 ribosomal protein is thought to enhance the affinity of ribosomes for mRNAs with 5′-terminal oligopyrimidine tracts, thus promoting translation initiation, and also has a role in regulating cell size [42]. Interestingly, convergence of ERK and mTOR signaling also occurs on another factor, eukaryotic translation initiation factor 4B (eIF4B). One study found that both ERK and mTOR phosphorylate eIF4B at the same residue (Ser422), which increases the protein's interaction with eukaryotic initiation factor 3 [44]. Our study shows that the VMR-resistant BCPAP cells when treated with the drug had over 9-fold higher proportion of the S6 ribosomal protein in the phosphorylated state compared to that of VMR-sensitive BCPAP cells (Figure 5B, 0.37:0.04). The rate of protein synthesis, thus translating to cell growth rate, was more than 9 times less suppressed by VMR in the resistant cells compared to in sensitive cells.

The pattern we observed in terms of activation of S6 ribosomal protein in our VMR-resistant BCPAP cells more closely resembles that seen with activation of mTOR compared to activation of ERK. While ERK was constitutively hyperactive within resistant BCPAP cells, activation of mTOR and S6 ribosomal protein was not increased in the resistant line, but less inhibited by VMR treatment compared to susceptible BCPAP. It is possible that the relatively refractory nature of S6 ribosomal protein activation in resistant cells is mediated by both ERK and mTOR, especially since activation of mTOR itself is inhibited by BRAFV600E inhibition in normal BCPAP. Hyperactive ERK may be contributing to the persistent mTOR phenotype.

Characterization of development of resistance also agrees with results of our prior study investigating combination therapy using BRAFV600E and mTOR inhibitors in thyroid cancer cells [36]. Earlier, we found that the combination reduced cell viability in resistant BCPAP cells. In addition, the combination had a greater effect on VMR-sensitive BCPAP cells in that mTOR inhibitors sensitized these cells to cytotoxic effects of VMR. Taken together, the data suggests that proteins capable of mediating resistance in the presence of increasing concentrations of a targeted inhibitor may also be indicated as targets in susceptible cells. Determining rational targets to be inhibited in combination with existing kinase inhibitors is a growing topic of interest due to the potential for limiting resistance, enhancing apoptosis and/or suspending tumor growth indefinitely. Within the RAF-mutated cancers, researchers found that the combination of sorafenib (RAF inhibitor) and flavopiridol (pan-CDK inhibitor) synergistically enhanced cytotoxicity in RAS/RAF mutant breast cancer cells [45]. In addition, treatment of sorafenib combined with the phytochemical fisetin (PI3K inhibitor) reduced tumor growth, inhibited proliferation and angiogenesis, and induced apoptosis in melanoma xenografts more effectively compared to each individual treatment [46]. Novel combination therapy is a concept that can be applied broadly among human cancers, is constantly evolving, and may provide a solution for targeted inhibitor resistance.

While this study focuses on protein expression and cell signaling within thyroid cells, research in melanoma has identified additional means of resistance through genetic mutation in other ERK proteins such as NRAS, alternative splice patterns of *BRAFV600E*, and gene amplification of *BRAFV600E* [25, 28–31]. It is possible that genetic mutation occurred concurrently with signaling changes in our thyroid cells. The plasticity of responses to targeted therapy suggests that while targeting critical signaling molecule BRAFV600E will provide immediate and potent antitumor effects, the eventual development of resistance must be combated with multifaceted inhibition. This inhibition may include multiple agents with different targets or agents which alter cellular phenotype more broadly.

## MATERIALS AND METHODS

### Cell culture

Thyroid cell lines used in this study include Nthy-ori 3-1 (immortalized normal thyroid cells), BCPAP (papillary thyroid cancer cells), and 8505c (anaplastic thyroid cancer cells). Dr. Norman L. Eberhardt (Mayo Clinic, Rochester, MN) generously gifted the Nthy-ori 3-1 cell line. BCPAP and 8505c cell lines were purchased from DSMZ in Braunschweig, Germany. All cell lines were cultured in Roswell Park Memorial Institute (RPMI)-1640 supplemented with 10% fetal bovine serum (FBS), penicillin 10,000 IU/mL, streptomycin 10,000 μg/mL, and 2 mM L-glutamine.

### Resistant BCPAP cell line

VMR-resistant BCPAP cells were created by intermittently treating BCPAP cells with increasing concentrations of VMR (Chemietek, Indianapolis, IN). Cells were subjected to treatment for 24 hour periods, subcultured and allowed to grow to 70% confluence before the next treatment. After each treatment period, a portion of cells were collected for analysis in terms of phospho-ERK expression. Resistant BCPAP cells were maintained in culture with intermittent treatments of 16 μM VMR.

### Alamar blue cell viability assay

Cells were plated at 4,000 cells per well in 96-well plates and allowed to adhere overnight. Complete media was replaced with 100 μL phenol-red-free RPMI containing 5% charcoal dextran-treated FBS and various concentrations of VMR including 0, 10, 100, 1000, 20000, and 50000 nM. Plates were incubated at 37°C for 96 hours and then 10 μL Alamar blue was added to each well. Plates were incubated for 4 hours protected from light before optical density was measured at 570 nm and 600 nm for control.

### Western blotting

Thyroid cells were grown to 70% confluence in T75 flasks and complete media was replaced with 5% charcoal dextran-treated FBS-containing media for 24 hours. Cells were treated with +/− 10 μM VMR for 1, 3, 6, 12, and 24 hours. For whole cell lysates, cells were collected by gentle scraping with cell scrapers, subjected to radioimmunoprecipitation assay buffer (50 mM Tris-HCl [pH 7.4], 150 mM NaCl, 0.2% sodium deoxycholate, 0.1% sodium dodecyl sulfate [SDS], 0.5% NP40, and 1 μM Pefabloc) and vortexed every 5 minutes for 30 minutes over ice. Lysates were centrifuged at 14,000 rpm for 30 minutes at 4°C and each supernatant was collected. Cytoplasmic extracts were obtained using NE-PER Nuclear and Cytoplasmic Extraction Reagents kit (Pierce). Volumes containing 10 μg or 20 μg protein per sample were subjected to 12% SDS-polyacrylamide gel electrophoresis. Proteins on gels were transferred to Immobilon-P membranes for 2 hours at 220 mA in a transfer chamber. Membranes were blocked with 5% milk in TBST (10 mM Tris-HCl, pH 7.5, 200 mM NaCl, 0.05% Tween-20) for 1.5 hours at room temperature and then incubated overnight in primary antibodies (Cell Signaling) at 4° C. Membranes were washed three times with TBST for 5 minutes per wash and incubated with secondary antibody for 2 hours at room temperature. After 4 TBST washes of 10 minutes each, membranes were developed by enhanced chemiluminescence (Thermo Scientific) and detected on X-ray film. All densitometry was performed using the ImageJ program.

### Immunoprecipitation

Thyroid cells were plated in T75 flasks and allowed to grow until 70% confluence. Complete media was replaced with 5% charcoal dextran-treated FBS-containing media and flasks were incubated for 24 hours. Cells were treated for an additional 24 hours with +/− 10 μM VMR. Cells were collected by gentle scraping, spun down, and subjected to 1% NP-40 lysis buffer on ice for 10 minutes with periodic mixing. Samples were centrifuged for 10 minutes at 14,000 rpm and 4°C and supernatants were collected. GammaBind Plus Sepharose beads (GE Healthcare) were washed 3 times with PBS. CRAF antibody (BD Bioscience) was added to beads and the mixture was incubated for 2 hours at 4°C with constant rotation. At the same time, 100 μg of sample lysate was also incubated for 2 hours at 4°C with constant rotation to pre-clear lysates. The beads coated with antibody were washed three times with PBS before the pre-cleared lysate was added to them. This mixture was incubated overnight in eppendorf tubes at 4°C with constant rotation. The tubes were centrifuged for 10 minutes at 14,000 rpm and beads were washed three times in 1% NP-40 lysis buffer. Loading buffer was added to the samples. Samples were boiled for ten minutes and spun down. Supernatants were subjected to 12% SDS-polyacrylamide gel electrophoresis as in our prior studies and probed for CRAF with primary antibody (Cell Signaling).
